# Retroperitoneal Lympathic Malformations as First Presentation of Lymphangioleiomyomatosis

**DOI:** 10.5334/jbsr.2468

**Published:** 2021-06-03

**Authors:** Christine Lenfant, Cristina Anca Dragean

**Affiliations:** 1 Cliniques universitaires Saint-Luc, BE

**Keywords:** lymphangioleiomyomatosis, retroperitoneum, cystic abdominal mass, lymphatic malformation, mediastinum

## Abstract

**Teaching Point:** This case highlights the extrapulmonary lymphatic abnormalities that may be associated with pulmonary lymphangioleiomyomatosis.

## Case study

A 37-year-old woman consulted in our hospital for an increase in the size of her left thigh and abdominal discomfort. Ultrasound excluded venous thrombosis the lower limb.

**Figure 1:**
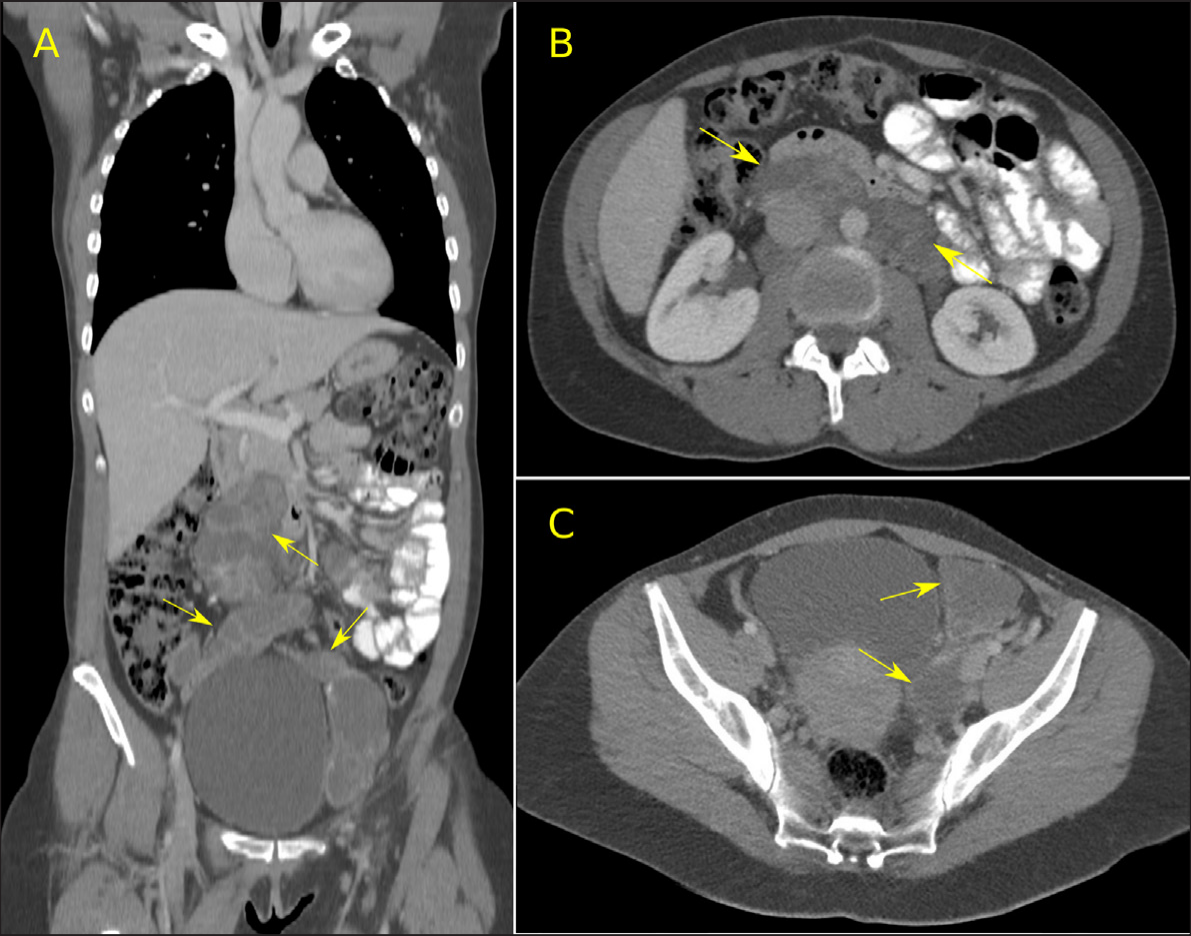


Contrast-enhanced body computed tomography (CT) was performed to exclude obstruction on the venous return. It showed two large retroperitoneal well-defined multi-locular cystic masses with homogeneous fluid content and enhancement of the cyst walls and septa (***Figure 1***), suggestive of lymphatic malformations.

The proximal mass surrounded the aorta and the inferior vena cava, spreading along the common iliac axes (***Figure 1A, B***, arrows). The left extraperitoneal pelvic mass (***Figure 1C***, arrows) pressed against the bladder and extended in the inguinal canal, likely causing the left thigh enlargement.

**Figure 2:**
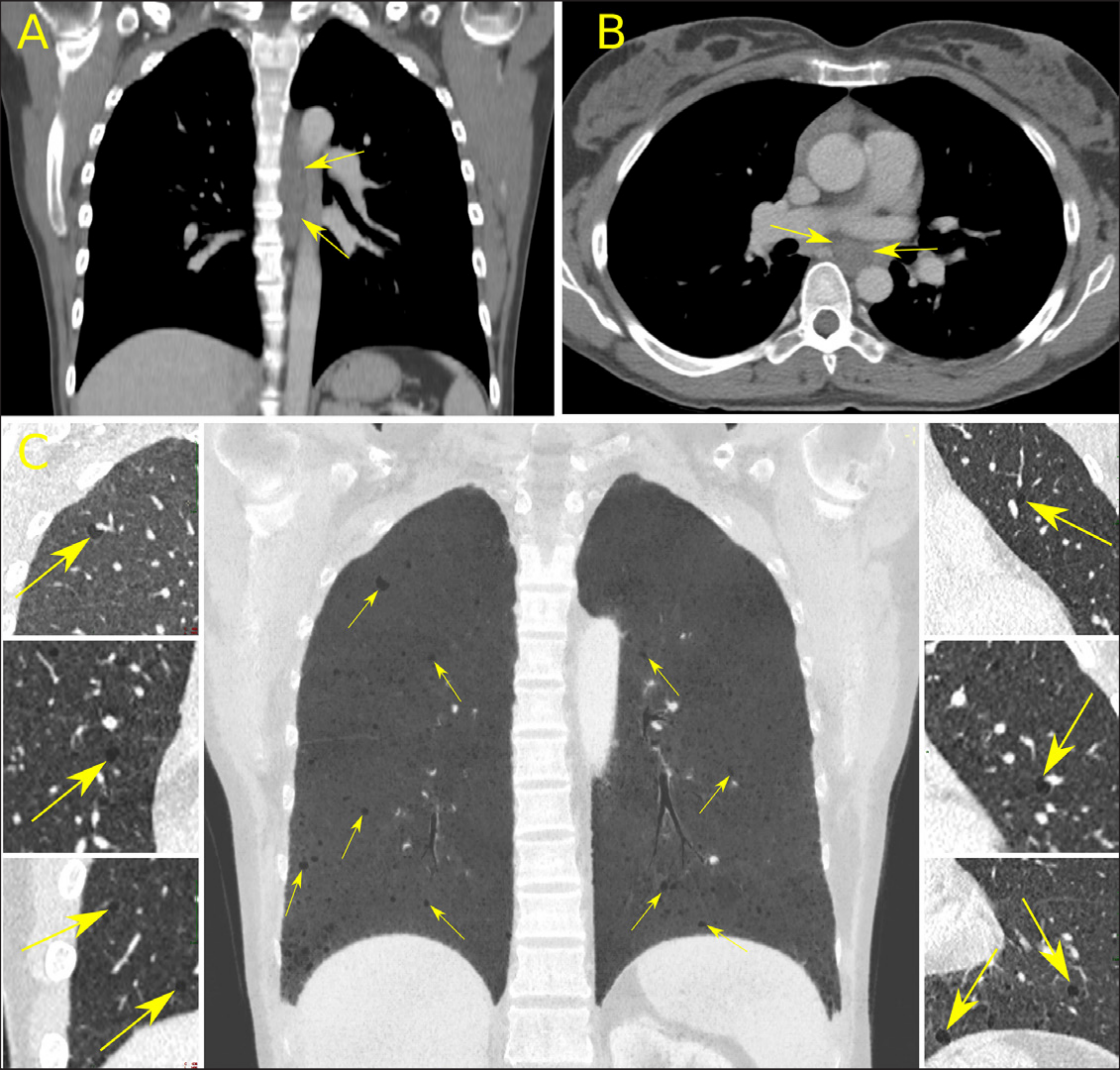


There was a similar low-attenuating mass in the posterior mediastinum (***Figure 2A, B***, arrows) and thin-walled cysts of uniform distribution and variable sizes in both lungs (***Figure 2C***, arrows) compatible with pulmonary lymphangioleiomyomatosis (LAM).

The abdominal lymphatic malformations were considered as lymphangioleiomyomas, an extra-pulmonary manifestation of LAM.

**Figure 3:**
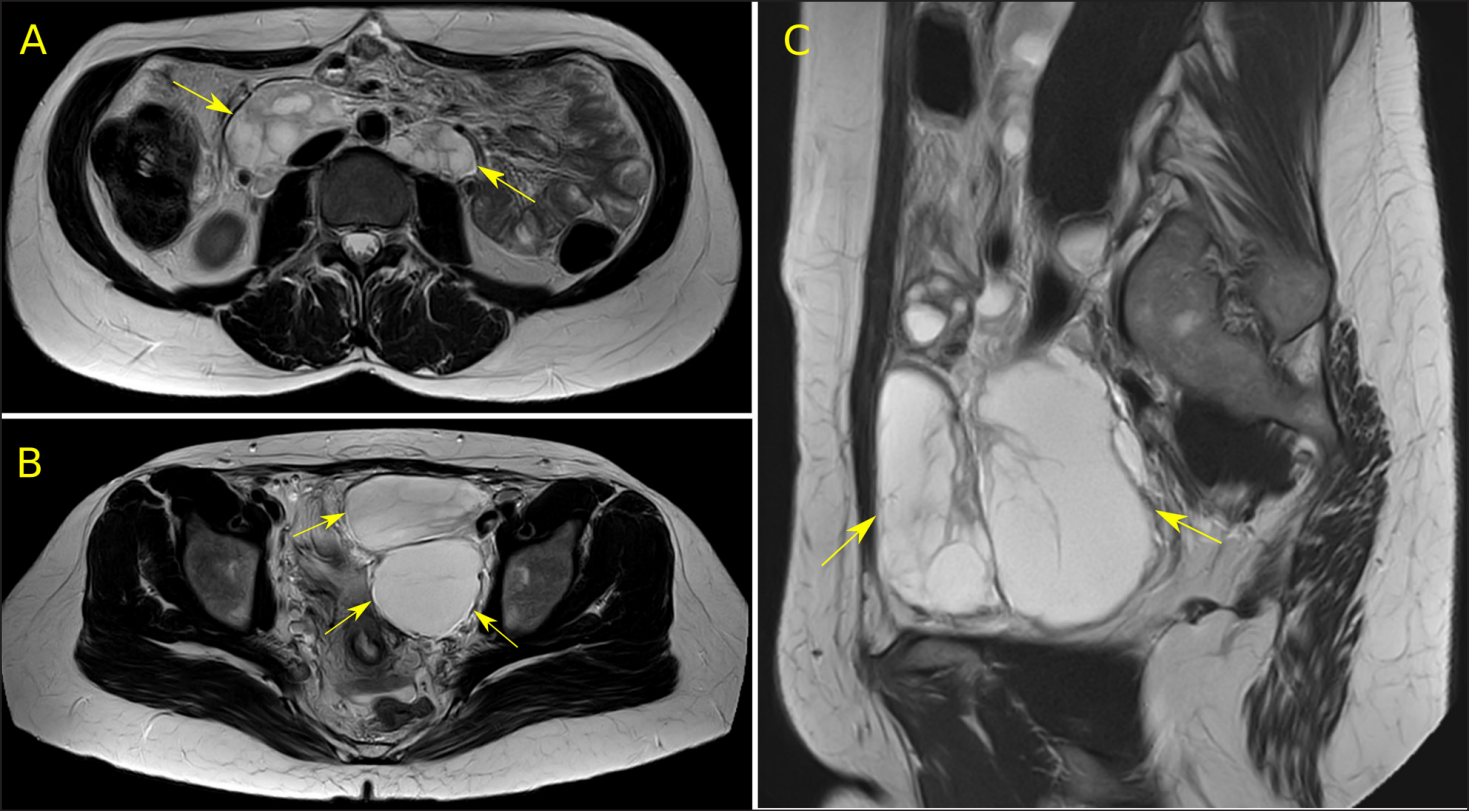


We further investigated the patient history. An MRI was actually performed previously in another hospital for abdominal pain which we had no knowledge of at the time of the CT. It showed the same retroperitoneal (***Figure 3A***) and pelvic (***Figure 3B, C***) masses.

After our diagnosis, our patient was treated with Sirolimus, stabilizing her lymphangioleiomyomas. However, they increased after a following pregnancy and sclerotherapy was recently proposed for the symptomatic pelvic lymphangioleiomyoma.

## Comment

LAM is a rare multisystem disorder that usually affects adult women, associated with cystic destruction of the lungs, abdominal neoplasm, and lymphatic thoraco-abdominal involvement. It can be associated with tuberous sclerosis complex.

Lymphatic involvement in LAM includes lymphangioleiomyomas and adenopathy, chylous ascites, and chylothorax. The lymphangioleiomyomas are oestrogen-dependant benign cystic masses, sometimes causing incomfort. Since LAM is a rare disease usually first presenting with dyspnea, there are no established guidelines concerning first lymphatic abdominal presentation. However, LAM diagnosis and accurate management is crucial since its prognosis is variable and can lead to pulmonary transplant [1]. Abdominal lymphatic malformations should therefore raise attention for a pulmonary LAM and should be discussed with the internist. A way of proceeding could be a referral for lung function testing or serum Vascular Endothelial Growth Factor-D (VEGF-D) testing which is usually elevated in case of LAM [1]. Based on these results, an High-Resolution Computed Tomography (HRCT) of the chest could be performed. Diagnosis of LAM can be based on adenopathy biopsy but biopsy is not necessary in case of characteristic or compatible lung HRCT combined with abdominal lymphatic manifestation [1].
